# The Relationship between Type 2 Diabetes Mellitus and Related Thyroid Diseases

**DOI:** 10.1155/2013/390534

**Published:** 2013-04-04

**Authors:** Chaoxun Wang

**Affiliations:** Department of Endocrinology, Shanghai Pudong Hospital, Fudan University Pudong Medical Center, 2800 Gongwei Road, Huinan Town, Pudong, Shanghai 201399, China

## Abstract

Type 2 diabetes mellitus (T2DM) has an intersecting underlying pathology with thyroid dysfunction. The literature is punctuated with evidence indicating a contribution of abnormalities of thyroid hormones to type 2 DM. The most probable mechanism leading to T2DM in thyroid dysfunction could be attributed to perturbed genetic expression of a constellation of genes along with physiological aberrations leading to impaired glucose utilization and disposal in muscles, overproduction of hepatic glucose output, and enhanced absorption of splanchnic glucose. These factors contribute to insulin resistance. Insulin resistance is also associated with thyroid dysfunction. Hyper- and hypothyroidism have been associated with insulin resistance which has been reported to be the major cause of impaired glucose metabolism in T2DM. The state-of-art evidence suggests a pivotal role of insulin resistance in underlining the relation between T2DM and thyroid dysfunction. A plethora of preclinical, molecular, and clinical studies have evidenced an undeniable role of thyroid malfunctioning as a comorbid disorder of T2DM. It has been investigated that specifically designed thyroid hormone analogues can be looked upon as the potential therapeutic strategies to alleviate diabetes, obesity, and atherosclerosis. These molecules are in final stages of preclinical and clinical evaluation and may pave the way to unveil a distinct class of drugs to treat metabolic disorders.

## 1. Introduction

The role of hyperthyroidism in diabetes was investigated in 1927, by Coller and Huggins proving the association of hyperthyroidism and worsening of diabetes. It was shown that surgical removal of parts of thyroid gland had an ameliorative effect on the restoration of glucose tolerance in hyperthyroid patients suffering from coexisting diabetes [1].

There is a deep underlying relation between diabetes mellitus and thyroid dysfunction [2]. A plethora of studies have evidenced an array of complex intertwining biochemical, genetic, and hormonal malfunctions mirroring this pathophysiological association [2, 3]. 5′ adenosine monophosphate-activated protein kinase (AMPK) is a central target for modulation of insulin sensitivity and feedback of thyroid hormones associated with appetite and energy expenditure [3]. Hypothyroidism (Hashimoto's thyroiditis) or thyroid over activity (Graves' disease) has been investigated to be associated with diabetes mellitus. A meta-analysis reported a frequency of 11% in thyroid dysfunction in the patients of diabetes mellitus [4]. Autoimmunity has been implicated to be the major cause of thyroid-dysfunction associated diabetes mellitus [5–7].

Unmanaged pro diabetes, both type 1 and type 2, may induce a “low T3 state” characterized by low serum total and free T3 levels, increase in reverse T3 (rT3) but near normal serum T4 and TSH concentrations [8]. The relation between T2DM and thyroid dysfunction has been a less explored arena which may behold answers to various facts of metabolic syndrome including atherosclerosis, hypertension, and related cardiovascular disorders.

T2DM owes its pathological origin to inappropriate secretion of insulin, due to defective islet cell function or beta cell mass. Continuous consumption of calories-rich meals, junk food and sedentary lifestyle have culminated into an epidemic of diabetes projected to afflict around 300 million people across the globe by 2020 [9]. Defective insulin secretion leads to various metabolic aberrations in T2DM, spanning from hyperglycemia due to defective insulin-stimulated glucose uptake and upregulated hepatic glucose production, along with dyslipidaemia, which includes impaired homeostasis of fatty acids, triglycerides, and lipoproteins [10].

## 2. Epidemiology

Thyroid dysfunction is a common endocrine disorder with variable prevalence. Wickham survey reveals that a prevalence of thyroid dysfunction in male adults in England was 6.6% [11]. According to Colorado prevalence study, 9.5% of participants were found to have elevated thyroid-stimulating hormone (TSH), while 2.2% had a low TSH. According to the National Health and Nutrition Examination Survey (NHANES III Study), hypothyroidism and hyperthyroidism were reported in 4.6% and 1.3% of the total participants [12], respectively. The prevalence of thyroid dysfunction is advancing with age all over the world, and frequency of prevalence was higher in women than men. The prevalence of subclinical hypothyroidism is reported to be about 4 to 8.5 percent, and may be as high as 20 percent in women older than 60 years. The prevalence of subclinical hyperthyroidism is reported to be approximately 2%.

The prevalence of thyroid disorder in diabetic population was reported to be 13.4% with higher prevalence (31.4%) in female T2DM patients as compared to (6.9%) in male T2DM patients [13]. The prevalence of thyroid dysfunction in T2DM patients was reported to be 12.3% in Greece and 16% in Saudi Arabia by Akbar et al. [14]. Considerably, T2DM patients were more prone to thyroid disorders.

## 3. Peripheral Effects of Thyroid Hormones on Insulin Secretion and Sensitivity

Thyroid hormones directly control insulin secretion. In hypothyroidism, there is a reduction in glucose-induced insulin secretion by beta cells, and the response of beta cells to glucose or catecholamine is increased in hyperthyroidism due to increased beta cell mass. Moreover, insulin clearance is increased in thyrotoxicosis [15, 16].

### 3.1. Thyrotoxicosis

**Figure 1:**
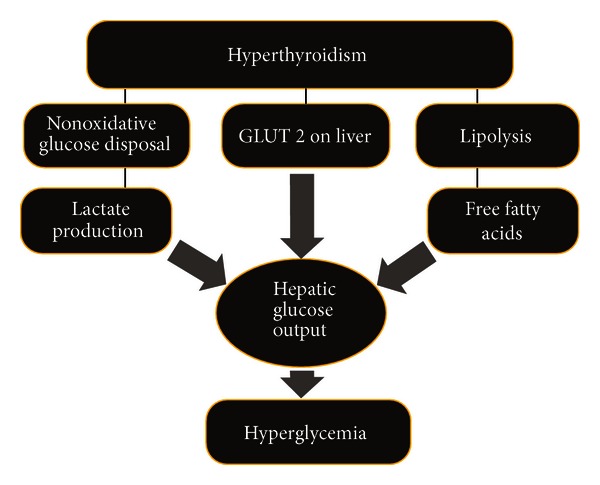
The relation between hyperthyroidism and hyperglycemia via lipid metabolism oxidative stress and hepatic dysfunction.

Increased glucose output from liver is the pivotal reason for the induction of hyperinsulinaemia, induction of glucose intolerance, and development of peripheral insulin resistance [17]. Glucose tolerance in thyrotoxicosis is caused by elevated hepatic glucose output along with upregulated glycogenolysis [2]. This phenomenon is responsible for worsening of subclinical diabetes and exaggeration of hyperglycaemia in T2DM. Thyrotoxicosis may lead to ketoacidosis also due to elevated lipolytic actions and increased hepatic *β* oxidation [18, 19]. This phenomenon has been shown in Figure 1.

### 3.2. Hypothyroidism

**Figure 2:**
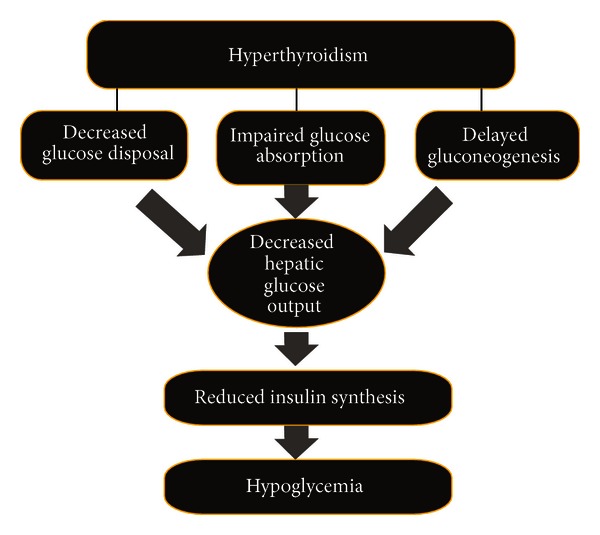
The relation between hypothyroidism and hypoglycemia mediated by reduced insulin synthesis and impaired hepatic glucose output.

Reduced glucose absorption from gastrointestinal tract accompanied by prolonged peripheral glucose accumulation, gluconeogenesis, diminished hepatic glucose output and reduced disposal of glucose are hallmarks of hypothyroidism [20]. In overt or subclinical hypothyroidism, insulin resistance leads to glucose-stimulated insulin secretion [2]. In subclinical hypothyroidism, diminished rate of insulin stimulated glucose transport rate caused by perturbed expression of glucose transporter type 2 gene (GLUT 2) translocation may lead to insulin resistance. Moreover, due to reduced renal clearance of insulin in hypothyroid conditions, physiological requirements of insulin were diminished. Anorectic conditions in hypothyroidism may also contribute to reduced insulin in this state. An enhanced dose of insulin is required to ameliorate hypothyroidism, but the therapy warrants caution for adrenal or pituitary failure [21]. This phenomenon has been shown in Figure 2. 

## 4. Relation between the Pathological Features Exhibited in Hyperthyroidism and T2DM: Role of Insulin Resistance and Other Factors 

The pathological features of T2DM include increased intestinal glucose absorption, reduced insulin secretion, and change in the *β*-cell mass [22]. Further, symptoms also include increased insulin degradation [23], increased glucagon secretion [24], increased hepatic glucose production [24], enhanced catecholamines, and insulin resistance [25]. These factors have been investigated to be an integral part of hyperthyroidism as well [26]. Hence, an intersection of pathological basis occurs which gives us cue to an array of physiological aberrations which are common in hyperthyroidism and T2DM. Among the above-mentioned symptomatology, insulin resistance has been the most important facet connecting thyroid dysfunction and T2DM. Insulin resistance is a condition which occurs in both hypothyroidism and hyperthyroidism [27].

Insulin resistance in the muscles and liver is a characteristic feature of T2DM. An undisturbed glucose homeostasis and intact insulin secretary response and unperturbed sensitivity of the tissues to insulin are essential to maintain normal blood glucose levels [28–31].

Glucose disposal is mediated by the conjoint effect of insulin and hyperglycemia to modulate three basic phenomenon. Firstly, diminution of endogeneous (hepatic) glucose production. Secondarily, enhanced uptake of glucose (hepatic and splanchnic). Thirdly, upregulation of glucose by peripheral tissues (skeletal muscles). Glucose uptake into muscles is modulated by glycolysis and glycogen synthesis. Hepatic insulin resistance is characterized by glucose overproduction inspite of fasting hyperinsulinemia, and enhanced rate of hepatic glucose output was the pivotal modulator of increased fasting plasma glucose (FPG) concentration in T2DM subjects [24]. In insulin resistance in the postabsorptive state, muscle glucose is upregulated but the efficiency of uptake is reduced. In the wake of such conditions, reduced glucose uptake into the muscles and enhanced hepatic glucose output lead to worsening of glucose metabolism. 

The term harmonious quartet is used to address the core pathology of insulin resistance [24]. Deregulated glucose disposal and metabolism in adipocytes, muscles, and liver, along with impaired insulin secretion by the pancreatic beta cells, constitute the four major organ system abnormalities which play a definitive role in the pathogenesis of T2DM. It is worth considering that insulin resistance has been a proven condition in hyperthyroidism as well as hypothyroidism. Insulin resistance also leads to impaired lipid metabolism according to recent findings [32]. Hence, it appears that insulin resistance is the possible link between T2DM and thyroid dysfunction.

Insulin resistance and *β* cell function are inversely correlated with thyroid stimulating hormone which may be explained by insulin-antagonistic effects of thyroid hormones along with an increase in TSH. The higher serum TSH usually corresponds to lower thyroid hormones via negative feedback mechanism. As TSH increased, thyroid hormones decreased and insulin antagonistic effects are weakened. These observations demonstrate that insulin imbalance is closely associated with thyroid dysfunction and the phenomenon id mediated via *β* cell dysfunction [33].

### 4.1. Association of Insulin Resistance in Hyperthyroidism and Subclinical Hyperthyroidism

Hyperthyroidism has been associated with insulin resistance which has been linked with elevated glucose turnover, increased intestinal glucose absorption, elevated hepatic glucose output, increased free fatty acid concentrations, increased fasting and or postprandial insulin an proinsulin levels, and increased peripheral glucose transport accompanied by glucose utilization [27, 34]. T2DM patients with thyroid dysfunction have been proven to be more susceptible to ketosis [35] and ketogenesis [36]. Insulin resistance has been shown to be associated with subclinical hypothyroidism, which is in turn linked to impaired lipid balance and risk of development of metabolic syndrome [37–39].

#### 4.1.1. Role of Liver

In hyperthyroidism, endogenous glucose production is elevated and reduces hepatic insulin sensitivity in humans [40] due to glycogenesis and glycogenolysis. The role of hypothalamus mediated sympathetic action in liver has been proposed [41] along with increased expression of GLUT 2 transporters in liver which ultimately lead to elevation in plasma free fatty acid [42, 43].

#### 4.1.2. Role of Muscles

There is marked increase in the skeletal glucose utilization in hyperthyroid state [34]. Increased glucose utilization has been reported to be mediated by insulin stimulated glucose oxidation rates [44–46]. Under such conditions, reduced glyco genesis has been reported due to insulin stimulated nonoxidative glucose disposal, which is accompanied by redirection of intracellular glucose towards glycolysis and lactate formation [27]. The transport of lactate from periphery to liver leads to enhanced production of glucose via Cori's cycle. Hyperthyroidism has also been associated with enhanced insulin sensitivity [47]. Increased peripheral insulin resistance has been coupled with elevated expression of bioactive inflammatory mediators including adipokines (IL-6 and TNF-alpha) [16] which lead to insulin resistance.

#### 4.1.3. Role of Fat Tissues

Haluzik et al. summarized that rate of local lipolysis in the abdominal subcutaneous adipose tissue was a result of modulation of norepinephrine (NE) levels and adrenergic postreceptor signaling by thyroid hormones [48]. Other studies reported that thyroid hormones are necessary for the mobilization of the tissue lipids especially brown adipose tissues (BATs) which are the fuel for the production of heat [49]. Hypothyroidism and decreased thyroid hormone level are responsible for decreased thermogenesis in BAT. S14 and lipogenesis are important factors for thermogenesis mediated by thyroid hormone [50].

### 4.2. Association of Insulin Resistance in Hypothyroidism and Subclinical Hypothyroidism

Insulin resistance has been shown to be caused in hypothyroidism in various *in vitro* and preclinical studies [51–53] where it was found that peripheral muscles became less responsive in hypothyroid conditions. A possible role of dysregulated metabolism of leptin has been implicated for such pathology [53]. A direct relation between hypothyroidism and insulin resistance has been demonstrated by various authors [15, 54–56]. 

Subclinical hypothyroidism has been reported to be associated with insulin resistance [55, 57, 58]. However, conflicting findings have also been reported by other workers [59, 60], indicating the need of further investigations in this domain.

## 5. Genetic Causes of T2DM and Thyroid Dysfunction: The Possible Intersection

### 5.1. Effect of Thyroid Hormones on the Liver: The Role of Genes

Various genes have been identified which are identified with gluconeogenesis, glycogen metabolism, and insulin signaling. These include glucose 6 phosphate, protein kinase B (Akt2), *β*_2_ adrenergic receptor, inhibitory G protein (Gi), phosphoenolpyruvate kinase (PEPCK) [25], pyruvate carboxylase (PC), GLUT 2 [42, 64], malic enzyme [65], and carbohydrate response element binding protein (ChREBP) [66]. A raised hepatic expression of GLUT 2 in hyperthyroid rats was observed as compared to hypothyroid rats [64].

Transcription of various enzymes involved in lipid metabolism has been reported to increase in hyperinsulinemic or insulin-resistant mice [2, 67]. Transcriptional activation of malic acid has proven to be involved in fatty acid synthesis [68].

### 5.2. Effect of Thyroid Hormones on the Skeletal Muscle

The various genes which influence the interaction of thyroid hormone and skeletal muscles include GLUT1, GLUT4 [64], *β*_2_ adrenergic receptors [69], phosphoglycerate kinase (PGK) [70], PPAR gamma coactivator-1 alpha (PGC-1 alpha) [71], and mitochondrial uncoupling protein [72]. Amongst the various genes identified, GLUT-4 and UCP-3 have been studied in detail. In the skeletal muscles, GLUT 4 has been proven to be mediated by the influence of T3, and it can elevate basal and insulin mediated transport of glucose [64]. 

Mitochondrial uncoupling protein 3 (UCP 3) is a recently identified gene and has been unveiled to be associated with glucose metabolism and decreased fatty acid oxidation [73]. It has also been reported to play a pivotal role in the downregulated activation of Akt/PKB and 5′ adenosine monophosphate-activated protein kinase signaling [2, 73]. The role of T2 has also been explored and it has been proven that it is associated with sarcolemmal GLUT-4. Phosphofructokinase and glycolytic enzymes have been associated with the T2-mediated GLUT 4 activity [74]. A number of genes have been associated with peripheral glucose metabolism [2].

Autoimmune causes are reported to be responsible for the genetic dysfunction in the diabetic patient suffering from thyroid related disorders. However, these findings advocate an immense clinical evidence to support association between T1DM (Type 1 diabetes mellitus) and autoimmune thyroid dysfunction (AITD) [75, 76]. Arrays of genes involved in metabolism of glucose are modulated by active thyroid hormone T3 by binding to the thyroid hormone receptors. These receptors are derived from TR**α**1, TR**β**1, TR**β**2, and TR**β**3. These are four major T3 binding isoforms [77]. TR**α**1 is hypothesized to regulate the metabolic effects of thyroid hormone. TR**β**1 and TR**β**2 are related with maintenance of hypothalamic-pituitary-thyroid axis and keeping the euthyroid state [78]. 

It has been investigated that 3,5,3-triiodothyronine is derived from T4. It can be activated via removing an iodine atom from the phenolic ring by the iodothyronine deiodinases type 1 (D1) and type 2 (D2). Type 3 deiodinase (D3) inactivates thyroid hormone by removing an iodine atom from the tyrosyl ring. The deiodinases are expressed in various tissues, and their expression levels vary enormously during development and are regulated by thyroid hormone status. Type deiodinase (D1) is expressed in liver, while type 2 deiodinase (D2) is expressed in adipose tissue and skeletal muscle. They are involved in regulation of bioavailability of T3 and hence, the response to insulin. Elevated concentrations of T3 are associated novel missense variant (Thr92Ala). This phenomenon is closely associated with insulin resistance. It is also associated with a surge in glucose turnover accompanied by an upregulation of insulin-mediated glucose disposal in skeletal muscle and adipose tissue. 

This phenomenon mediated via positive regulation of insulin sensitive GLUT-4 transcription [78, 79] showed that there were profound genomic effects of T3 on hepatic glucose metabolism. TR expressed in the hepatocyte and stimulation of T3-sensitive neurons in the hypothalamus-modulated hepatic glucose production via sympathetic projections to the liver are mediated by circulating glucoregulatory hormones [79]. Recent findings have elucidated polymorphism of deiodinase type 2 (DIO2) gene, Thr92Ala, which suggest homozygosity for this polymorphism which in turn is responsible for enhanced risk of T2DM [80]. 

A separate meta-analysis indicated that intracellular tri-iodothyronine (T3) is responsible for aberrations in insulin sensitivity [4]. It has also been reported that polymorphism of Thr92Ala leads to a lower activity of type 2 deiodinase which in turn is associated with insulin resistance. The D2 gene has a peculiar transcriptional and posttranslational regulation. It is a potential modulator of insulin action in skeletal muscle and adipose tissue through the regulation of the GLUT-4 gene transcription [81].

Investigations using skeletal muscles in hypothyroid and euthyroid humans have revealed a discernable influence on the downregulated expression of glucose transporter 5 (GLUT 5) but not GLUT 4 [57, 82]. Glucose oxidation and glycogen synthesis are reduced in hypothyroidism [21]. Simultaneous increase in the insulin sensitivity occurs when the levels of thyroid hormone were increased. This phenomenon is governed by intracellular generation of T3 as polymorphisms of DIO2 with reduced T3 generation and also contributes to insulin resistance [80]. In hyperthyroidism, the expression of GLUT 2 is increased as compared to euthyroid state [16]. In such conditions, perturbations in lipid metabolism further link TH to insulin resistance [16]. Thyroid hormone causes elevation in the plasma fatty acid levels in hyperthyroid conditions but not in hypothyroid conditions. Low intracellular fatty acid levels are associated with hepatic insulin sensitivity via modulation of cellular insulin uptake or lipid oxidation [83]. Fatty acid uptake mediated by TH is a tissue-specific phenomenon and is upregulated in both hypo, and hyperthyroidism [79].

**Figure 3:**
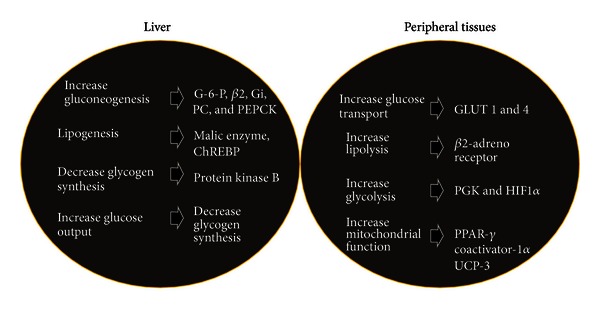
: Effect of thyroid hormones on the liver and peripheral tissues.

Thyrotoxicosis leads to enhanced lipid peroxidation whereas hypothyroidism causes diminished glucose oxidation. LDL clearance leads to lowered cholesterol and triglyceride levels. TH instigates upregulation of catecholamine action leading to lipolysis on adipocytes and enhancement of circulating FA. Elevated supply of FA counteracts TH-mediated elevated hepatic long-chain FA oxidative process. Elevated circulating FA levels and availability of gluconeogenic substrates from peripheral reserves reciprocates increased gluconeogenesis in T3-treated animals. It has been reported that T3 enhances fasting plasma glucose and free FA levels. Activation of peripheral substrates explains precipitation of hyperglycemia in thyrotoxicosis [84]. Paradoxically, hyperglycemic effect of thyrotoxicosis can be reversed by increased blood supply to muscles providing a better supply of substrate [16]. This phenomenon is shown in Figure 3.

## 6. Relation of Antidiabetic Therapy (Metformin) and Risk of Thyroid Related Perturbations

Cappelli et al. evaluated the thyroid hormone profile by studying the interaction between metformin and circulating thyroid function parameters in patients who were started on metformin [85]. A pilot study on diabetic hypothyroid patient revealed baseline reduction of TSH level after 6 months; similarly a large cohort study on diabetic patients showed significant fall of TSH level in euthyroid patients on L-T4 substitution and subclinical hypothyroid patients who did not receive LT4 treatment, except in euthyroid patients after 1 year on metformin. This study concluded that TSH lowering effect of metformin only seen in untreated hypothyroid patient and with L-T4 replacement therapy irrespective of thyroid function test. Similar findings were reported by Vigersky et al., Cappelli et al., and Chen et al. [86–88].

*In vitro* studies support the use of metformin in other thyroid diseases other than hypothyroidism. Metformin has inhibited the cell proliferation and growth-stimulatory effect of insulin on thyroid carcinoma cell lines. Same study showed the stimulation of apoptosis and enhancement in the action of chemotherapeutic agents (doxorubicin and cisplatin) by metformin [89]. Other reports support growth inhibitory effect of metformin in mammalian cell lines mediated by mammalian target of Rapamycin (mTOR) and cyclin D1 [90].

## 7. Therapeutic Role of Thyroid Hormone Analogues

Thyroid hormones have profound influence on various physiological processes ranging from metabolism of lipid, protein, and carbohydrate. The literature is punctuated with reports claiming antiatherogenic and lipolytic influences of thyroid hormones. However, their deleterious effects on bone, muscles, and heart are major hurdles [91]. Thyroid hormone analogues have paved the way for the development of novel strategies in the treatment of atherosclerosis, diabetes and obesity [91]. Recent investigations and subsequent findings have provided many cues that could behold trails of complex physiological mechanisms in the endocrine crosstalk of glycaemic surge and thyroid dysfunction [91].

Development of potent thyroid hormone analogues that selectively elude the harmful effects of thyroid hormone, and at the same time, produce desirable therapeutic effects has been the cynosure of scientific research [92–94]. The thrust of the research has been in designing TH analogues which are devoid of the cardiac complications [27, 95]. Preclinical investigations have demonstrated that carbohydrate response element-binding protein (ChREBP) is the pivotal transcription factor modulating the stimulation of hepatic lipogenesis mediated by glucose. It is the primary target of thyroid hormones in liver and white adipose tissues [66]. ChREBP has been reported to be regulated by TR*β* only and not TR*α* in liver and white adipose tissue [66].

## 8. Clinical Guidelines Governing the Role of Detection of Thyroid Detection in T2DM Patients

Various studies have been undertaken to understand the role, importance, and need of determination of thyroid dysfunction in the patients of T2DM. It has been unequivocally apparent that testing for thyroid dysfunction in T2DM patients is necessary and should be carried out annually [13]. Guidelines for screening of thyroid in diabetes patients in UK and USA are presented in Table 1.

The “American Thyroid Association” guidelines for T2DM patients require frequent testing for thyroid dysfunction. They recommend testing from 35 years of age, and every 5 years thereafter in adults. High-risk patients may require more frequent testing. The American Association of Clinical Endocrinologists, Thyroid Disease Clinical Practice Guidelines (2002) recommends thyroid palpation and TSH in diagnosis, especially if goitre or other autoimmune disease presents in association with T2DM. Regular screening for thyroid abnormalities in all diabetic patients will allow early treatment of subclinical thyroid dysfunction. A sensitive serum TSH assay is the screening test of choice. It has also been proposed that in T2DM patients, a TSH assay should be performed at diagnosis and then repeated at least every 5 years.

## 9. Conclusion

In internal medicine, it is repeatedly proven that the association between thyroid dysfunction and diabetes mellitus is evident. Thyroid dysfunction chiefly comprises hypothyroidism and hyperthyroidism although the entity belongs to the same organ but with vast difference in pathophysiology as well as clinical picture. The interface between thyroid malfunction owing to diabetes is a matter of investigation. The literature suggests that polyendocrinal multidysfunction leads to stimulation of a cascade of reactions which are actually antihomeostatic in nature. For instance, hypoadrenalism as well as hypopituitarism exhibits strong linkage with hypothyroidism and consequently diabetes mellitus. 

Recent findings have evidenced the intricate bond between subclinical hypothyroidism and diabetes mellitus that deceptively contribute to the major complications such as retinopathy and neuropathy. Cardiovascular events and micro- or macro-angiopathies are the counterreflection of resurgence of heavily disturbed lipid metabolism due to thyroid dyscrasias. It is also evident from the existing literature that insulin resistance bears an indispensable role in connecting T2DM and thyroid dysfunction. Novel molecules have shown the path for the development of suitable thyroid hormone receptor analogues to treat metabolic diseases. It is important to diagnose thyroid dysfunction in T2DM patients, and this practice should be inculcated in clinical settings with immediate effect to nourish further understanding of thyroid dysfunction and T2DM.

## Figures and Tables

**Table 1:** Diabetic practice guidelines for thyroid screening in patients with diabetes.

Sr. no.	Guidelines	Type 2 diabetes	Comments
(1)	American Thyroid Association guidelines for detection of thyroid dysfunction [61]	Patients with diabetes may require more frequent testing	Recommends TSH from 35 yrs, and every 5 yrs thereafter in all adults; high risk persons may require more frequent tests Diabetes mentioned as high-risk but does not distinguish between T1DM and T2DM

(2)	American Association of Clinical Endocrinologists, Thyroid disease clinical Practice guidelines, 2002 [62]	Thyroid palpation and TSH at diagnosis and at regular intervals, especially if goitre or other autoimmune disease presents	No specific recommendation for T2DM

(3)	British Thyroid Association and Association of Clinical Biochemistry Guidelines, 2006 [63]	TFT at baseline but routine annual TFT is not recommended	TSH and antibodies are recommended in diabetic patients in pregnancy and postpartum
